# Supplementary material for: Clustering trees: a visualization for evaluating clusterings at multiple resolutions
Source: Gigascience. 2018 Jul 11;7(7):giy083. doi: 10.1093/gigascience/giy083 (PMC6057528; doi:10.1093/gigascience/giy083)
Supplement: GIGA-D-18-00086_Revision_1.pdf [file giy083_giga-d-18-00086_revision_1.pdf]

GigaScience

Clustering trees: a visualisation for evaluating clusterings at multiple resolutions

--Manuscript Draft--

**Manuscript Number:**	GIGA-D-18-00086R1
**Full Title:**	Clustering trees: a visualisation for evaluating clusterings at multiple resolutions
**Article Type:**	Research
**Funding Information:**	Department of Education, Australian Governement — Mr Luke Zappia<br>National Health and Medical Research Council (APP1126157) — Dr Alicia Oshlack
**Abstract:**	Clustering techniques are widely used in the analysis of large data sets to group together samples with similar properties. For example, clustering is often used in the field of single-cell RNA-sequencing in order to identify different cell types present in a tissue sample. There are many algorithms for performing clustering and the results can vary substantially. In particular, the number of groups present in a data set is often unknown and the number of clusters identified by an algorithm can change based on the parameters used. To explore and examine the impact of varying clustering resolution we present clustering trees. This visualisation shows the relationships between clusters at multiple resolutions allowing researchers to see how samples move as the number of clusters increases. In addition, meta-information can be overlaid on the tree to inform the choice of resolution and guide in identification of clusters. We illustrate the features of clustering trees using a series of simulations as well as two real examples, the classical iris dataset and a complex single-cell RNA-sequencing dataset. Clustering trees can be produced using the clustree R package available from CRAN (https://CRAN.R-project.org/package=clustree) and developed on GitHub (https://github.com/lazappi/clustree).
**Corresponding Author:**	Alicia Oshlack<br><br>AUSTRALIA
**Corresponding Author Secondary Information:**	
**Corresponding Author's Institution:**	
**Corresponding Author's Secondary Institution:**	
**First Author:**	Luke Zappia
**First Author Secondary Information:**	
**Order of Authors:**	Luke Zappia<br>Alicia Oshlack
**Order of Authors Secondary Information:**	
**Response to Reviewers:**	Reviewer #1<br>The authors in the manuscript try to answer an important and biologically relevant question. The manuscript is written well and the message is clearly explained. However, we have some concerns<br>and comments on the manuscript.<br><br>1.The presented method is conceptually equivalent to visualisation of hierarchical clustering, only applicable to other clustering methods. This should be made more clear in the text.<br><br>We have mentioned the relationship to hierarchical clustering in the paper and discussed the differences between this and clustering trees. While we accept the similarities between them we believe that clustering trees are significantly different,

Powered by Editorial Manager® and ProduXion Manager® from Aries Systems Corporation

both in how they are constructed and how they would be used.

2.We think more datasets should be considered in the study.

We have added an additional section that uses five simulated datasets to illustrate what clustering trees would look like in different scenarios based on a suggestion from reviewer 3. We believe that this is useful in helping to explain the concepts presented in the paper. Adding more real datasets would provide extra examples but in our opinion would not convey the messages of the manuscript with more clarity.

3.Clustertree considers cluster stability measured across ks. Cluster stability is not a novel concept and the authors should include an brief overview of the existing literature on cluster stability in the introduction (e.g. Ben-Hur et al. 2002, Luxburg 2010) and explain how their method is different from the existing approaches.

Thank you for the suggestion and the references. We had added a paragraph that mentions the concept of cluster stability more generally.

4.In application to scRNAseq the elements of the clustering tree are methodologically very similar to the cluster stability index introduced in the SC3 package (https://www.nature.com/articles/nmeth.4236). It would be good to have a comparison of the two methods.

We had not considered the SC3 stability index before and there are indeed similarities, particularly as both clustering trees and the SC3 measure can be produced from just a set of clustering labels. We believe this measure could be useful for users and have implemented this method in the clustree package. The SC3 stability is now automatically calculated for each cluster and can be used to colour the nodes of the tree. Examples of this are included in the simulation section and the differences discussed.

5.(major) It is not obvious (at least for us) to understand from the clustering tree which k is the best. Even for a simple iris dataset it was hard for me to guess that k=3 is the right k. Maybe there are too many colours in the tree picture. Could the authors provide an algorithmic approach to suggest the appropriate k(s) based on the tree perhaps in conjunction with some kind of metadata laid over the tree?

We intend clustering trees to be a tool that can help make the decision of which resolution to use, but not one that can provide a concrete suggestion. This could have been made clearer in the previous version and we have tried to do so in our revised text. Adding the simulation examples gives the reader a much clearer demonstration of what can happen to a clustering tree as a dataset becomes over-clustered. We have also tried to emphasise that clustering trees become more useful when combined with other metrics or domain knowledge and that they provide a new way to visualise this information across resolutions.

Reviewed by Tallulah Andrews and Vladimir Kiselev
Reviewer #2
The paper presents a new method to construct clustering trees for single-cell RNA-seq. While I recognize the task is very important due to the emerging importance of single-cell technologies, the proposed method only contains incremental improvements. Before addressing the following concerns I have, I would not recommend acceptance.

We do not believe the reader has understood the point of this paper at all which is why they are recommending a rejection. We are not presenting a new clustering method. Our direct responses to the points in this review are below but we do not believe this a suitable review for this work.

Main concerns:

1.Clarity. This paper proposed a simple clustering method for ScRNA-seq. However, the difference to many other clustering method (e.g., hierarchical clustering) is not clearly stated. The novelty is not clear to me.

We do not propose a new clustering method but instead a new method for visualising the results of existing clustering methods across resolutions. This is discussed in the paper. We also mention that clustering trees could be used in any field that makes use of clustering, not just scRNA-seq analysis.

2.Validity. The paper constructs a hierarchical clustering tree without considering the specific characters of sparsity and high dropouts of single-cell RNA-seq. Due to the existence of drop-out, traditional Euclidean/correlation metrics are not reliable (See "Visualization and analysis of single-cell RNA-seq data by kernel-based similarity learning", Nature Methods, 2017). However, this paper did not provide any specific solution to this problem. I am wondering why this method is particularly suitable for single-cell RNA-seq.

Our method is not designed specifically for scRNA-seq data and is in fact independent of any type of dataset or clustering method. As explained in our response to the previous point we propose a method for visualising clustering results, not a new clustering method.

3.Experiments. This paper applies the proposed methods on one simulation and one real PBMC dataset. However, no comparisons with other methods is provided. It is very hard to judge how well the proposed method is really performing. Visualization is also hard to judge. The lack of detailed experiments and comparisons is the main concern before acceptance.

The submitted version of the manuscript did not consider any simulated datasets but provided examples based on the real iris and PBMC datasets. Simulated datasets have been added in the revised manuscript. We do not believe there is an existing visualisation that is directly comparable but we have included the SC3 stability index as an example of an existing cluster stability measure.

4.References: This paper is missing a few important references about single-cell anlaysis: For instance: "Revealing the vectors of cellular identity with single-cell genomics", Nature Biotech., 2016

As our paper is not specifically about scRNA-seq data or analysis we do not feel the need to reference all important papers in that field. We have provided an introduction to scRNA-seq data that is designed to help a general reader understand the PBMC dataset and why clustering would be useful in that setting. We believe this is sufficient for a technique that could be applied to many fields.
Reviewer #3:
Identification of the suitable number of clusters is an age-old question in clustering analysis. Standard methods for identifying the number of clusters make use of information about the 'tightness' of the clusters and the stability of the clusters with respect to some parameters. In this manuscript, Zappia and Oshlack present a new visualisation approach to explore the stability of cluster at different resolutions using a polytree visual representation, which allows for overlap of information of individual features and other external knowledge. This is an intuitive and powerful visualisation approach which I believe will be of widespread applications. I think this is a clever application of the hierarchical graph drawing technique. The manuscript is well written. I believe this manuscript is of value to the community.

However, I want to make the following suggestions:
Major:
-In figure 3 and figure 4, there are number of cases where a node has two parents. In almost all cases, the child node is placed under the parent node with the smallest node numbering instead of the node with the highest 'in-proportion' edge. For example, in Figure 4, the polytree has two nodes with two parent nodes. In both cases, the child node is placed below the parent node with the smaller 'in-proportion'. I thought it would make more sense to place them with the parent node with the higher 'in-proportion'.

We agree that this is a problem and it is the result of using existing layout algorithms which do not consider weight of edges in any way, sometimes resulting in layouts which seem to favour less important edges. We have addressed this by using only a subset of important edges (those with the greatest in-proportion for each node) to

	construct the layout. This simple modification is now the default setting in the clustree packages and results in more attractive tree which address the concerns you raise.<br><br>-Two 'positive' examples are described in the manuscript. I think it would be instructive to showcase what the resulting visualisation may look like if the clustering was performed on data with no or little underlying clustering structure. Could your visualisation identify 'bad' clustering results? For example, would the clustering tree of an entirely randomly generated data set looks differently from a data set with a strong clustering structure? A simulation study could be instructive here.<br><br>Thank you for the suggestion of adding a simulation study. We have added a new section to the paper that show some simulated scenarios. As you have suggested two of these are “null” examples including randomly generated uniform noise or a single cluster. We believe that these are instructive for the reader in showing what trees look like in different situations and how nodes and edges change as datasets are over-clustered.<br><br>-There are a number of graph drawing techniques for polytree, can the authors briefly review these methods and explain why the Reingold-Tilford or the Sugiyama layout was used?<br><br>These layout algorithms were chosen as they are the two methods designed for tree-like graphs available in the igraph package. We have added a paragraph to the manuscript that briefly explains how these algorithms work and why they were chosen.<br>Minor:<br>-It is important to point out that technically your 'tree' is a polytree, which is also called a directed acyclic graph. I do not object to calling it a 'tree' for simplicity throughout the manuscript, but I think it should be clearly noted in the introduction.<br>Thank you for introducing us to the idea of a polytree, this is not a term we had heard of before. You are correct that this is the graph structure produced by our algorithm and we have mentioned that in the text.
**Additional Information:**	
**Question**	**Response**
Are you submitting this manuscript to a special series or article collection?	No
**Experimental design and statistics**<br><br>Full details of the experimental design and statistical methods used should be given in the Methods section, as detailed in our Minimum Standards Reporting Checklist. Information essential to interpreting the data presented should be made available in the figure legends.<br><br>Have you included all the information requested in your manuscript?	Yes
**Resources**<br><br>A description of all resources used, including antibodies, cell lines, animals and software tools, with enough information to allow them to be uniquely identified, should be included in the Methods section. Authors are strongly encouraged to cite Research Resource	Yes

Identifiers (RRIDs) for antibodies, model organisms and tools, where possible.<br><br>Have you included the information requested as detailed in our Minimum Standards Reporting Checklist?	
**Availability of data and materials**<br><br>All datasets and code on which the conclusions of the paper rely must be either included in your submission or deposited in publicly available repositories (where available and ethically appropriate), referencing such data using a unique identifier in the references and in the “Availability of Data and Materials” section of your manuscript.<br><br>Have you have met the above requirement as detailed in our Minimum Standards Reporting Checklist?	Yes

Click here to download Manuscript clustree_paper_20180522[1].docx

Clustering trees: a visualisation for evaluating clusterings at multiple resolutions

Luke Zappia (1, 2)

Alicia Oshlack (1, 2)

1 Bioinformatics, Murdoch Children's Research Institute; 2 School of Biosciences, University of Melbourne

Clustering techniques are widely used in the analysis of large data sets to group together samples with similar properties. For example, clustering is often used in the field of single-cell RNA-sequencing in order to identify different cell types present in a tissue sample. There are many algorithms for performing clustering and the results can vary substantially. In particular, the number of groups present in a data set is often unknown and the number of clusters identified by an algorithm can change based on the parameters used. To explore and examine the impact of varying clustering resolution we present clustering trees. This visualisation shows the relationships between clusters at multiple resolutions allowing researchers to see how samples move as the number of clusters increases. In addition, meta-information can be overlaid on the tree to inform the choice of resolution and guide in identification of clusters. We illustrate the features of clustering trees using a series of simulations as well as two real examples, the classical iris dataset and a complex single-cell RNA-sequencing dataset. Clustering trees can be produced using the clustree R package available from CRAN (https://CRAN.R-project.org/package=clustree) and developed on GitHub (https://github.com/lazappi/clustree).

Keywords: Clustering - Visualisation - scRNA-seq

Introduction

Clustering analysis is commonly used to group similar samples across a diverse range of applications. Typically, the goal of clustering is to form groups of samples that are more similar to each other than to samples in other groups. While fuzzy or soft clustering approaches assign each sample to every cluster with some probability, and hierarchical clustering forms a tree of samples, most methods form hard clusters where each sample is assigned to a single group. This goal can be achieved in a variety of ways, such as by considering the distances between samples (e.g. *k*-means [1–3], PAM [4]), areas of density across the dataset (e.g. DBSCAN [5]) or relationships to statistical distributions [6].

In many cases the number of groups that should be present in a dataset is not known in advance and deciding the correct number of clusters to use is a significant challenge. For some algorithms, such as k-means clustering, the number of clusters must be explicitly provided. Other methods have parameters that, directly or indirectly, control the clustering resolution and therefore the number of clusters produced. While there are methods and statistics (such as the elbow method [7] or silhouette plots [8]) designed to help analysts decide which clustering resolution to use, they typically produce a single score which only considers a single set of samples or clusters at a time.

An alternative approach would be to consider clusterings at multiple resolutions and examine how samples change groupings as the number of clusters increases. This has lead to a range of cluster stability measures [9], many of which rely on clustering of perturbed or sub-sampled datasets. For example, the model explorer algorithm sub-samples a dataset multiple times, clusters each sub-sampled dataset at various resolutions and then calculates a similarity between clusterings at the same resolution to give a distribution of similarities which can inform the choice of resolution [10]. One cluster stability measure that isn't based on perturbations is that contained in the SC3 package for clustering single-cell RNA-sequencing data [11]. Starting with a set of cluster labels at different resolutions each cluster is scored, with clusters awarded increased stability if they share the same samples as a cluster at another resolution, but penalised for being at a higher resolution.

A similar simple approach is taken by the clustering tree visualisation we present here, without calculating scores: (i) a dataset is clustered using any hard clustering algorithm at multiple resolutions, producing sets of cluster nodes, (ii) the overlap between clusters at adjacent resolutions is used to build edges, (iii) the resulting graph is presented as a tree. This tree can be used to examine how clusters are related to each other, which clusters are distinct and which are unstable. In the following sections we describe how we construct such a tree and present examples of trees built from a classical clustering dataset and a complex single-cell RNA-sequencing (scRNA-seq) dataset. The figures shown here can be produced in R using our publicly

available clustree package. Although clustering trees can not directly provide a clustering resolution to use they can be a useful tool for exploring and visualising the range of possible choices.

Building a clustering tree

To build a clustering tree, we start with a set of clusterings allocating samples to groups at several different resolutions. These could be produced using any hard-clustering algorithm that allows control of the number of clusters in some way. For example, this could be a set of samples clustered using k-means with $k = 1,2,3$ as shown in Figure 1. We sort these clusterings so that they are ordered by increasing resolution (k), then consider pairs of adjacent clusterings. Each cluster $c_{k,i}$ (where $i = 1,...,n$ and n is the number of clusters at resolution k) is compared with each cluster $c_{k+1,j}$ (where $j = 1,...,m$ and m is the number of clusters at resolution $k + 1$). The overlap between the two clusters is computed as the number of samples that are assigned to both $c_{k,i}$ and $c_{k+1,j}$. We next build a graph where each node is a cluster and each edge is an overlap between two clusters. While we refer to this graph as a tree in this paper for simplicity it can more correctly be described as a polytree, a special case of a directed acyclic graph where the underlying undirected graph is a tree [12].

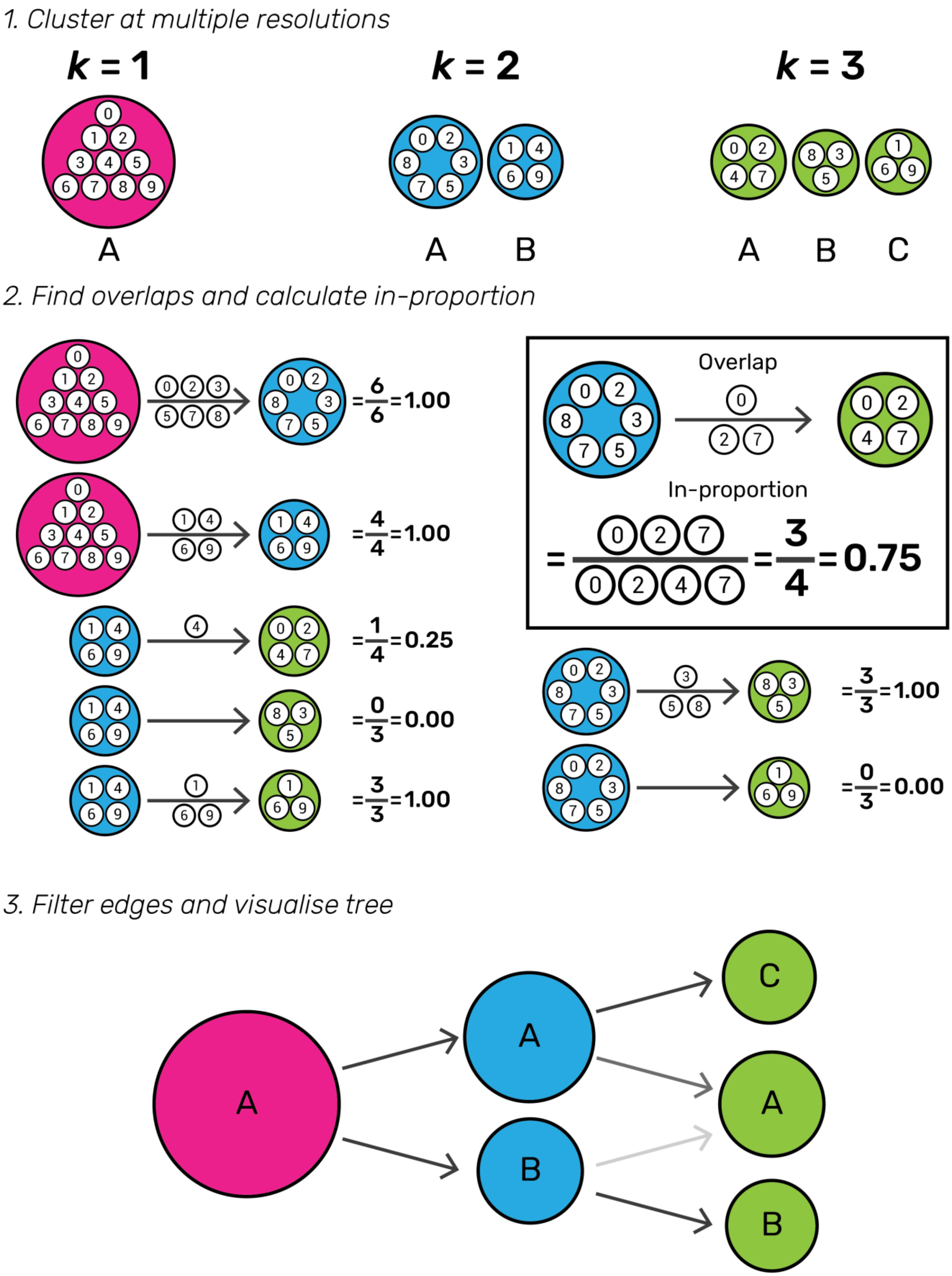

Figure 1 Illustration of the steps required to build a clustering tree. First a dataset must be clustered at different resolutions. The overlap in samples between clusters at adjacent resolutions is computed and used to calculate the in-proportion for each edge. Finally the edges are filtered and the graph visualised as a tree.

Many of the edges will be empty, for example in Figure 1 no samples in Cluster A at $k = 2$ end up in Cluster B at $k = 3$. In some datasets there may also be edges that contain few samples. These edges are not informative and result in a cluttered tree. An obvious solution for removing uninformative, low-count edges is to filter them using a threshold on the number of samples they represent. However, in this case the count of samples is not the correct statistic to use because it favours edges at lower resolutions and those connecting larger clusters. Instead we define the in-proportion metric as the ratio between the number of samples on the edge and the number of samples in the cluster it goes towards. This metric shows the importance of the edge to the higher resolution cluster independently of the cluster size. We can then apply a threshold to the in-proportion in order to remove less informative edges.

The final graph can then be visualised. In theory any graph layout algorithm could be used but for the clustree package we have made use of the two algorithms specifically designed for tree structures available in the igraph package [13]. These are the Reingold-Tilford tree layout, which places parent nodes above their children [14], and the Sugiyama layout which places nodes of a directed acyclic graph in layers while minimising the number of crossing edges [15]. Both of these algorithms can produce attractive layouts and as such we have not found the need to design a specific layout algorithm for clustering trees. By default the clustree package uses only a subset of edges when constructing a layout, specifically the highest in-proportion edges for each node. We have found that this often leads to more interpretable visualisations, however users can choose to use all edges if desired.

Whichever layout is used the final visualisation places the cluster nodes in a series of layers where each layer is a different clustering resolution and edges show the transition of samples through those resolutions. Edges are coloured according to the number of samples they represent and the in-proportion metric is used to control the edge transparency, highlighting more important edges. By default, the size of nodes is adjusted according to the number of samples in the cluster and their colour indicates the clustering resolution. The clustree package also includes options for

controlling the aesthetics of nodes based on the attributes of samples in the clusters they represent as shown in the following examples.

While a clustering tree is conceptually similar to the tree produced through hierarchical clustering there are some important differences. The most obvious are that a hierarchical clustering tree is the result of a particular clustering algorithm and shows the relationships between individual samples while the clustering trees described here are independent of clustering method and show relationships between clusters. The branches of a hierarchical tree show how the clustering algorithm has merged samples. In contrast, edges in a clustering tree show how samples move between clusters as the resolution changes and nodes may have multiple parents. While it is possible to overlay information about samples on a hierarchical tree this is not commonly done but is a key feature of the clustree package and how clustering trees could be used in practice.

A demonstration using simulations

To demonstrate what a clustering tree can look like in different situations and how it behaves as a dataset is over-clustered we present some illustrative examples using simple simulations (see methods). We present five scenarios: random uniform noise (Simulation A), a single cluster (Simulation B), two clusters (Simulation C),three clusters (Simulation D) and four clusters (Simulation E). Each cluster consists of 1000 samples (points) generated from a 100 dimensional normal distribution and each synthetic dataset has been clustered using k-means clustering with $k = 1,\dots,8$. We then use the clustree package to produce clustering trees for each dataset (Figure 2).

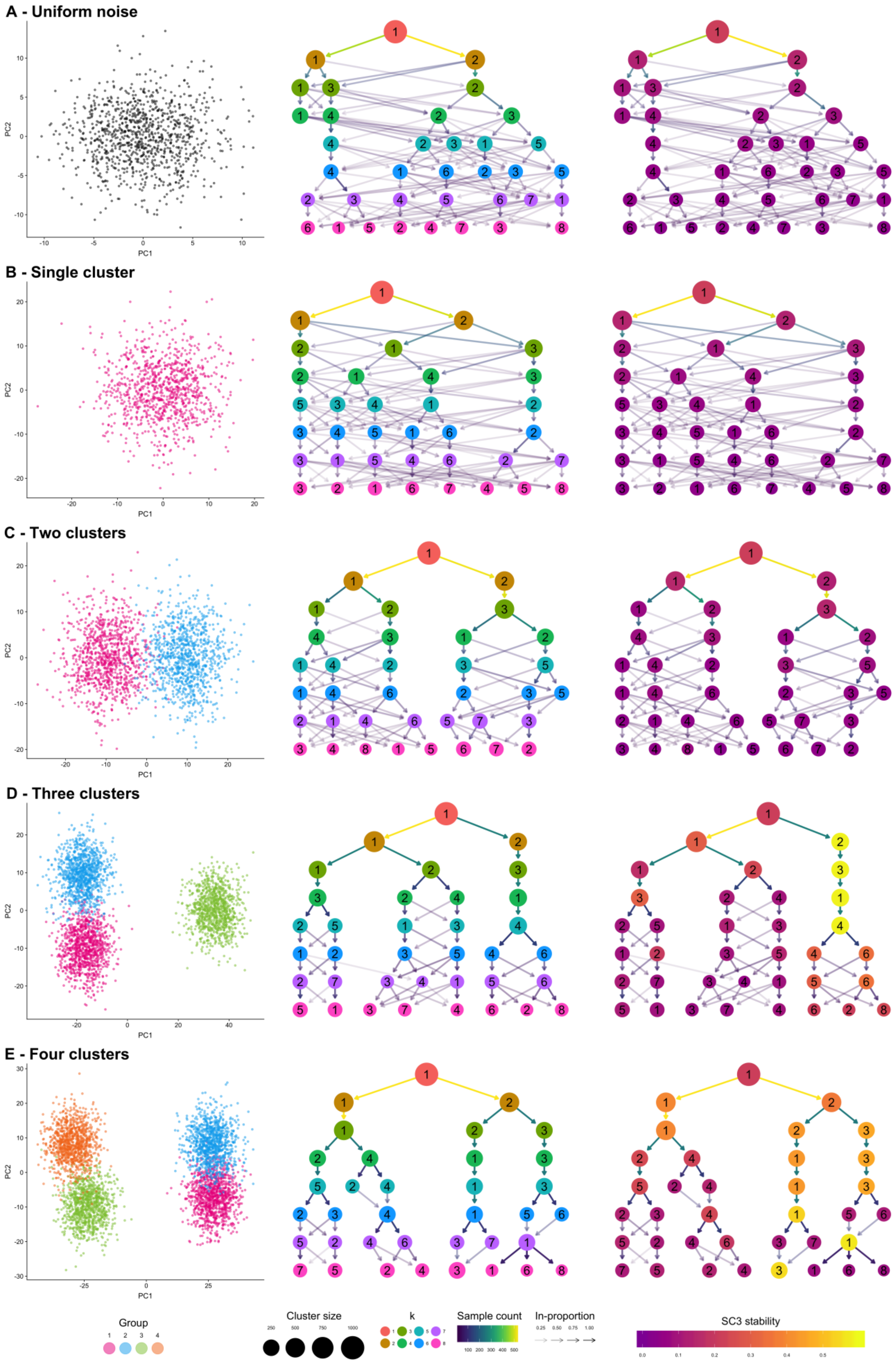
A - Uniform noise
B - Single cluster
C - Two clusters
D - Three clusters
E - Four clusters
PC1
PC2
Group
Cluster size
k
Sample count
In-proportion
SC3 stability

Figure 2 Five synthetic datasets used to demonstrate clustering trees. For each dataset a scatter plot of the first two principal components, a default clustering tree and and clustering tree with nodes coloured by the SC3 stability index from purple (lowest) to yellow (highest) are shown. The five datasets contain: A) random uniform noise, B) a single cluster, C) two clusters, D) three clusters and E) four clusters.

Looking at the first two examples (uniform noise (Figure 2A) and a single cluster (Figure 2B)) we can clearly see how a clustering tree behaves when a clustering algorithm returns more clusters than are truly present in a dataset. New clusters begin to form from multiple existing clusters and many samples switch between branches of the tree resulting in low in-proportion edges. Unstable clusters may also appear then disappear as the resolution increases as seen in Figure 2E. As we add more structure to the datasets the clustering trees begin to form clear branches and low in-proportion edges tend to be confined to sections of the tree. By looking at which clusters are stable and where low in-proportion edges arise we can infer which areas of the tree are likely to be the result of true clusters and which are caused by over-clustering.

The second clustering tree for each dataset shows nodes coloured according to the SC3 stability index for each cluster. As we would expect in the first two examples no cluster receives a high stability score. However, while we clearly see two branches in the clustering tree for the two cluster example (Simulation C) this is not reflected in the SC3 scores. No cluster receives a high stability score, most likely due to the high number of samples moving between clusters as the resolution increases. As there are more true clusters in the simulated datasets the SC3 stability scores become more predictive of the correct resolution to use, however it is important to look at the stability scores of all clusters at a particular resolution as taking the highest individual cluster stability score could lead to the incorrect resolution being used, as can be seen in the four cluster example (Simulation E). These examples show how clustering trees can be used to display existing clustering metrics in a way that can help to inform parameter choices.

A simple example

To further illustrate how a clustering tree is built, we will work through an example using the classical iris dataset [16]. This dataset contains measurements of the sepal length, sepal width, petal length and petal width from 150 iris flowers, 50 from each of three species: *Iris setosa*, *Iris*

versicolor and *Iris virginica*. The iris dataset is commonly used as example for both clustering and classification problems with the *Iris setosa* samples being significantly different to, and linearly separable from, the other samples. We have clustered this dataset using k-means clustering with $k = 1,...,5$ and produced the clustering tree shown in Figure 3A.

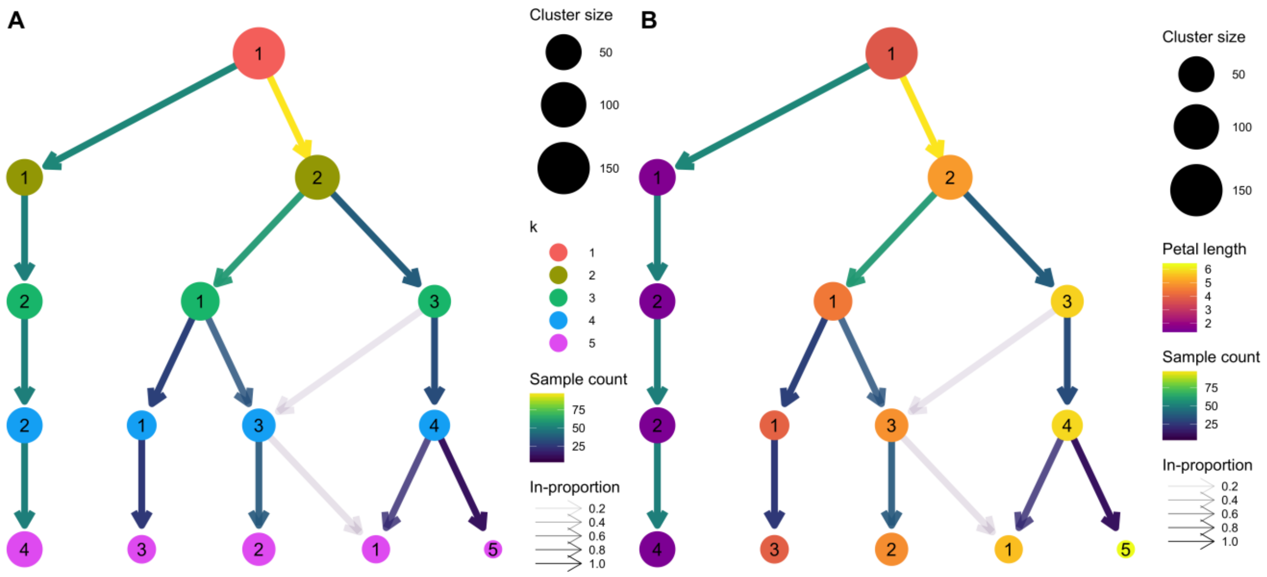

Figure 3 Clustering trees based on k-means clustering of the iris dataset. In A, nodes are coloured according to the value of k and sized according to the number of samples they represent. Edges are coloured according to the number of samples (from blue representing few to yellow representing many) and the transparency adjusted according to the in-proportion, with stronger lines showing edges that are more important to the higher resolution cluster. Cluster labels are randomly assigned by the k-means algorithm. B shows the same tree with the node colouring changed to show the mean petal length of the samples in each cluster.

We see that there is one branch of the tree that is clearly distinct (presumably representing *Iris setosa*), remaining unchanged regardless of the number of clusters. On the other side we see the cluster at $k = 2$ cleanly splits into two clusters (presumably *Iris versicolor* and *Iris virginica*) at $k = 3$ but as we move to $k = 4$ and $k = 5$ we see clusters being formed from multiple branches with more low in-proportion edges. As we have seen in the simulated examples, this kind of pattern can indicate that the data has become over-clustered and we have begun to introduce artificial groupings.

We can check our assumption that the distinct branch represents the *Iris setosa* samples and the other two clusters at $k = 3$ are *Iris versicolor* and *Iris virginica* by overlaying some known information about the samples. In Figure 3B we have coloured the nodes by the mean petal length

of the samples they contain. We can now see that clusters in the distinct branch have the shortest petals, with Cluster 1 at $k = 3$ having an intermediate length and Cluster 3 the longest petals. This feature is known to separate the samples into the expected species with *Iris setosa* having the shortest petals on average, *Iris versicolor* an intermediate length and *Iris virginica* the longest.

Although this is a very simple example it highlights some of the benefits of viewing a clustering tree. We get some indication of the correct clustering resolution by examining the edges and we can overlay known information to assess the quality of the clustering. For example, if we observed that all clusters had the same mean petal length it would suggest that the clustering has not been successful as we know this is an important feature that separates the species. We could potentially learn more by looking at which samples follow low proportion edges or overlaying a series of features to try and understand what causes particular clusters to split.

Clustering trees for single-cell RNA-seq data

One field that has begun to make heavy use of clustering techniques is the analysis of single-cell RNA-sequencing (scRNA-seq) data. Single-cell RNA-sequencing is a recently developed technology that can measure how genes are expressed in thousands to millions of individual cells [18]. This technology has been rapidly adopted in fields like developmental biology and immunology where it is valuable to have information from single cells rather than measurements that are averaged across the many different cells in a sample using older RNA sequencing technologies. One of the key uses for scRNA-seq is to discover and interrogate the different cell types present in a sample of a complex tissue. In this situation, clustering is typically used to group similar cells based on their gene expression profiles. Differences in gene expression between groups can then be used to infer the identity or function of those cells [19]. The number of cell types (clusters) in an scRNA-seq dataset can vary depending on factors such as the tissue being studied, its developmental or environmental state and the number of cells captured. Often the number of cells types is not known before the data is generated and some samples can contain

dozens of clusters. Therefore, deciding which clustering resolution to use is an important consideration in this application.

As an example of how clustering trees can be used in the scRNA-seq context we consider a commonly used Peripheral Blood Mononuclear Cell (PBMC) dataset. This dataset was originally produced by 10x Genomics and contains 2700 peripheral blood monocuclear cells, representing a range of well-studied immune cell types [20]. We have analysed this dataset using the Seurat package [21], a commonly used toolkit for scRNA-seq analysis, following the instructions in their tutorial with the exception of varying the clustering resolution parameter from zero to five (see methods). Seurat uses a graph-based clustering algorithm and the resolution parameter controls the partitioning of this graph, with higher values resulting in more clusters. The clustering trees produced from this analysis are shown in Figure 4.

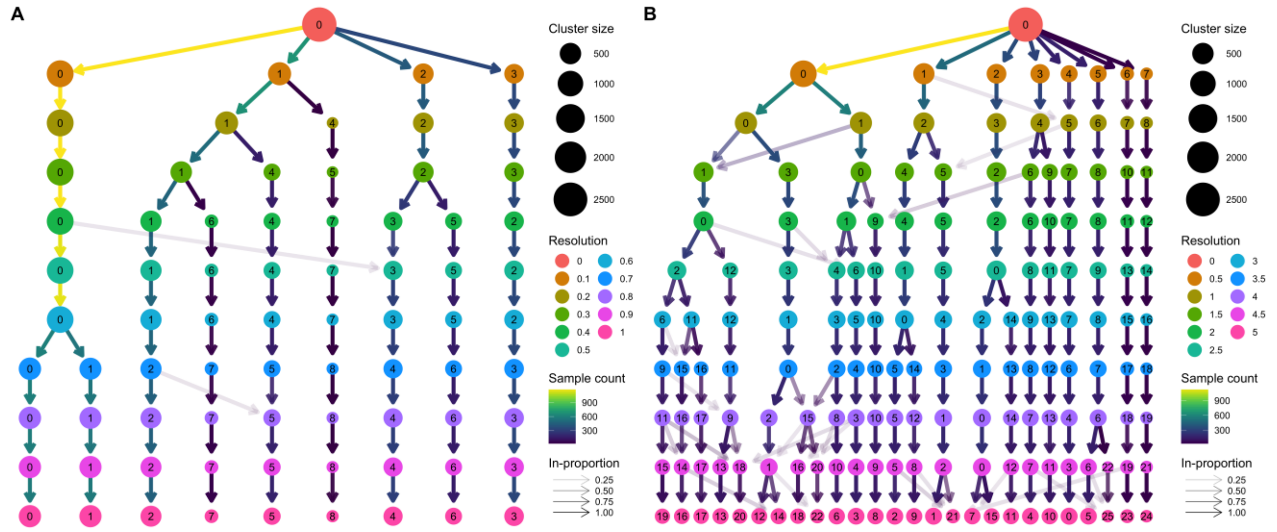

Figure 4 Two clustering trees of a dataset of 2700 Peripheral Blood Mononuclear Cells (PBMCs). A) results from clustering using Seurat with resolution parameters from zero to one. At a resolution of 0.1 we see the formation of four main branches, one of which continues to split up to a resolution of 0.5, after which there are only minor changes. B) resolutions from zero to five. At the highest resolutions we begin to see many low in-proportion edges indicating cluster instability. Seurat labels clusters according to their size with Cluster 0 being the largest.

The clustering tree covering resolutions zero to one in steps of 0.1 (Figure 4A) shows that four main branches form at a resolution of just 0.1. One of these branches, starting with Cluster 3 at resolution 0.1, remains unchanged while the branch starting with Cluster 2 splits only once at a resolution of 0.4. Most of the branching occurs in the branch starting with Cluster 1 which

consistently has sub-branches split off to form new clusters as the resolution increases. There are two regions of stability in this tree; at resolution 0.5-0.6 and resolution 0.7-1.0 where the branch starting at Cluster 0 splits in two.

Figure 4B shows a clustering tree with a greater range of resolutions, from zero to five in steps of 0.5. By looking across this range we can see what happens when the algorithm is forced to produce more clusters than are likely to be truly present in this dataset. As over-clustering occurs we begin to see more low in-proportion edges and new clusters forming from multiple parent clusters. This suggests that those areas of the tree are unstable and that the new clusters being formed are unlikely to represent true groups in the dataset.

Known marker genes are commonly used to identify the cell types that specific clusters correspond to. Overlaying gene expression information onto a clustering tree provides an alternative view that can help to indicate when clusters containing pure cell populations are formed. Figure 5 shows the PBMC clustering tree in Figure 4A overlaid with the expression of some known marker genes.

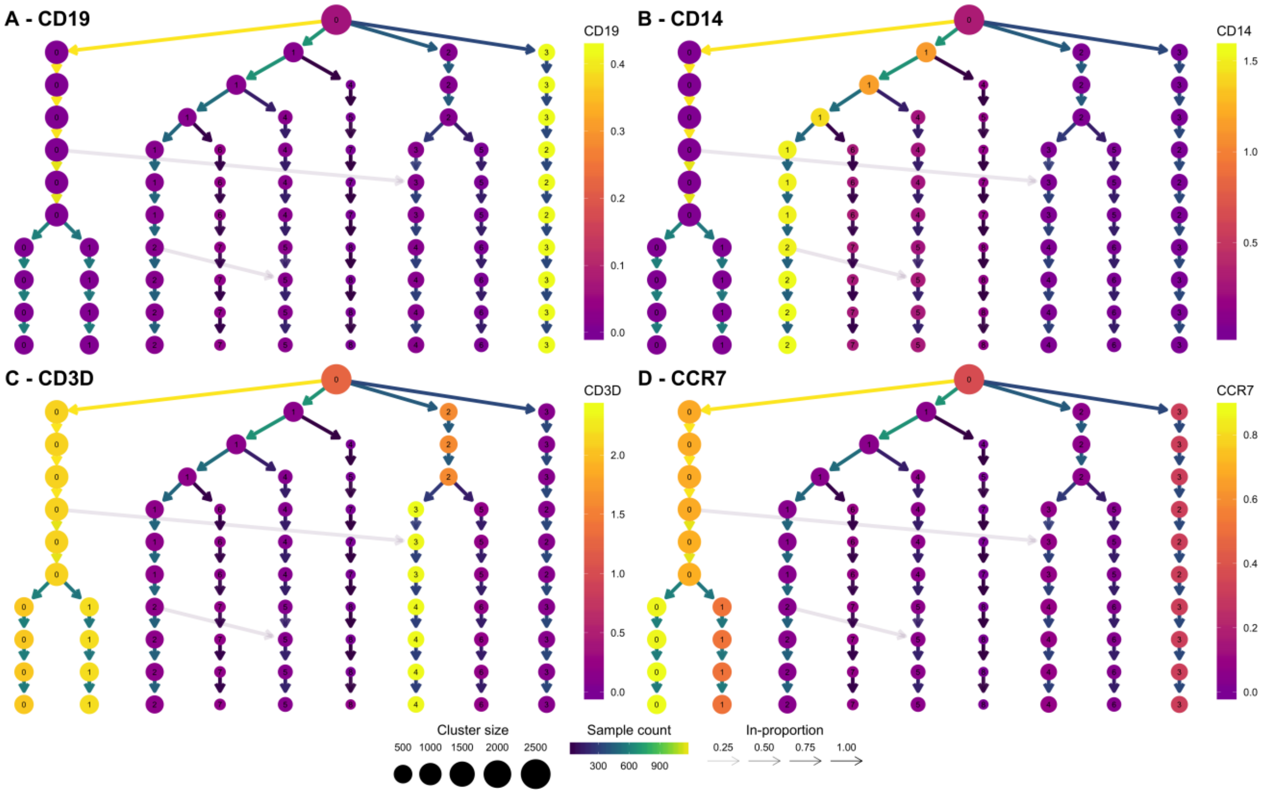

Figure 5 Clustering trees of the PBMC dataset coloured according to the expression of known markers. The node colours indicate the average of the log_2 gene counts of samples in each cluster. CD19 (A) identifies B cells, CD14 (B) shows a population of monocytes, CD3D (C) is a marker of T cells and CCR7 (D) shows the split between memory and naive CD4 T cells.

By adding this extra information, we can quickly identify some of the cell types. CD19 (Figure 5A) is a marker of B cells and is clearly expressed in the most distinct branch of the tree. CD14 (Figure 5B) is a marker of a type of monocyte, which becomes more expressed as we follow one of the central branches, allowing us to see which resolution identifies a pure population of these cells. CD3D (Figure 5C) is a general marker of T cells and is expressed in two separate branches, one which splits into low and high expression of CCR7 (Figure 5D), separating memory and naive CD4 T cells. By adding expression of known genes to a clustering tree, we can see if more populations can be identified as the clustering resolution is increased and if clusters are consistent with known biology. For most of the Seurat tutorial a resolution of 0.6 is used, but the authors note that by moving to a resolution of 0.8, a split can be achieved between memory and naive CD4 T cells. This is a split that could be anticipated by looking at the clustering tree with the addition of prior information.

Discussion and conclusion

Clustering similar samples into groups is a useful technique in many fields, but often analysts are faced with the tricky problem of deciding which clustering resolution to use. Traditional approaches to this problem typically consider a single cluster or sample at a time and may rely on prior knowledge of sample labels. Here we present clustering trees, an alternative visualisation that shows the relationships between clusterings at multiple resolutions. While clustering trees cannot directly suggest which clustering resolution to use they can be a useful tool for helping to make that decision, particularly when combined with other metrics or domain knowledge.

Clustering trees display how clusters are divided as resolution increases, which clusters are clearly separate and distinct, which are related to each other and how samples change groups as more clusters are produced. Although clustering trees can appear similar to the trees produced from hierarchical clustering there are several important differences. Hierarchical clustering considers

the relationships between individual samples and doesn't provide an obvious way to form groups. In contrast, clustering trees are independent of any particular clustering method and show the relationships between clusters, rather than samples, at different resolutions, any of which could be used for further analysis.

To illustrate the uses of clustering trees we presented a series of simulations and two examples of real analyses, one using the classical iris dataset and a second based on a complex scRNA-seq dataset. Both examples demonstrate how a clustering tree can help inform the decision of which resolution to use and how overlaying extra information can help to validate those clusters. This is of particular use to scRNA-seq analysis as these datasets are often large, noisy and contain an unknown number of cell types or clusters.

Even when determining the number of clusters is not a problem, clustering trees can be a valuable tool. They provide a compact, information dense, visualisation that can display summarised information across a range of clusters. By modifying the appearance of cluster nodes based on attributes of the samples they represent, clusterings can be evaluated and identities of clusters established. Clustering trees potentially have applications in many fields and in the future could be adapted to be more flexible, such as by accommodating fuzzy clusterings. There may also be uses for more general clustering graphs to combine results from multiple sets of parameters or clustering methods.

Methods

clustree

The clustree software package is built for the R statistical programming language. It relies on the ggraph package (https://github.com/thomasp85/ggraph), which is itself built on the ggplot2 [22] and tidygraph packages (https://github.com/thomasp85/tidygraph). Clustering trees are displayed using the Reingold-Tilford tree layout or the Sugiyama layout, both available as part of the igraph package.

Simulations

Simulated datasets were constructed by generating points from statistical distributions. The first simulation (Simulation A) consists of 1000 points randomly generated from a 100 dimensional space using a uniform distribution between zero and 10. Simulation B consists of a single normally distributed cluster of 1000 points in 100 dimensions. The centre of this cluster was chosen from a normal distribution with mean zero and standard deviation 10. Points were then generated around this centre from a normal distribution with mean equal to the centre point and a standard deviation of five. The remaining three simulations were produced by adding additional clusters. In order to have a known relationship between clusters the centre for the new clusters was created by manipulating the centres of existing clusters. For Cluster 2 a random 100 dimensional vector was generated from a normal distribution with mean zero and standard deviation two and added to the centre for Cluster 1. Centre 3 was the average of Centre 1 and Centre 2 plus a random vector from a normal distribution with mean zero and standard deviation five. To ensure a similar relationship between clusters 3 and 4 as between clusters 1 and 2, Centre 4 was produced by adding half the vector used to produce Centre 2 to Centre 3 plus another vector from a normal distribution with mean zero and standard deviation two. Points for each cluster were generated in the same way as for Cluster 1. Simulation C consists of the points in clusters 1 and 2, Simulation D consists of clusters 1, 2 and 3, Simulation E consists of clusters 1, 2, 3 and 4. Each simulated dataset was clustered using the “kmeans” function in the stats package with values of k from one to eight, a maximum of 100 iterations and 10 random starting positions. The clustering tree visualisations were produced using the clustree package with the tree layout. The simulated datasets and the code use to produce them are available from the repository for this paper (https://github.com/Oshlack/clustree-paper).

Iris dataset

The iris dataset is available as part of R. We clustered this dataset using the “kmeans” function in the stats package with values of k from one to five. Each value of k was clustered with a maximum of 100 iterations and with 10 random starting positions. The clustree package was used to

visualise the results using the Sugiyama layout. The clustered iris dataset is available as part of the clustree package.

PBMC dataset

The PBMC dataset was downloaded from the Seurat tutorial page (http://satijalab.org/seurat/pbmc3k_tutorial.html) and this tutorial was followed for most of the analysis. Briefly cells were filtered based on the number of genes they express and the percentage of counts assigned to mitochondrial genes. The data was then log-normalised and 1838 variable genes identified. Potential confounding variables (number of unique molecular identifiers and percentage mitochondrial expression) were regressed from the dataset before performing principal component analysis on the identified variable genes. The first 10 principal components were then used to build a graph which was partitioned into clusters using Louvain modularity optimisation [23] with resolution parameters in the range zero to five, in steps of 0.1 between zero and one and then in steps of 0.5. Clustree was then used to visualise the results using the tree layout.

Declarations

Ethics

Not applicable.

Availability of data and materials

The clustree package (RRID: SCR_016293) is available from CRAN (https://CRAN.R-project.org/package=clustree) and is being developed on GitHub at https://github.com/lazappi/clustree. The code and datasets used for the analysis in this paper are available from https://github.com/Oshlack/clustree-paper. The clustered iris dataset is included as part of clustree and the PBMC dataset can be downloaded from the Seurat tutorial page (http://satijalab.org/seurat/pbmc3k_tutorial.html) or the paper GitHub repository.

Competing interests

The authors declare no competing interests.

Funding

Luke Zappia is supported by an Australian Government Research Training Program (RTP) Scholarship. Alicia Oshlack is supported through a National Health and Medical Research Council Career Development Fellowship APP1126157. MCRI is supported by the Victorian Government's Operational Infrastructure Support Program.

Acknowledgements

Thank you to Marek Cmero for providing comments on a draft of the manuscript.

References

1. Forgy WE. Cluster analysis of multivariate data : efficiency versus interpretability of classifications. Biometrics [Internet]. 1965;21:768–9. Available from: https://ci.nii.ac.jp/naid/10009668881/

2. Macqueen J. Some methods for classification and analysis of multivariate observations. In 5th Berkeley Symposium on Mathematical Statistics and Probability [Internet]. 1967. Available from: http://citeseer.ist.psu.edu/viewdoc/summary?doi=10.1.1.308.8619

3. Lloyd S. Least squares quantization in PCM. IEEE Trans Inf Theory [Internet]. 1982;28:129–37. Available from: http://dx.doi.org/10.1109/TIT.1982.1056489

4. Kaufman L, Rousseeuw PJ. Partitioning Around Medoids (Program PAM). Finding Groups in Data [Internet]. John Wiley & Sons, Inc. 1990. pp. 68–125. Available from: http://dx.doi.org/10.1002/9780470316801.ch2

5. Ester M, Kriegel H-P, Sander J, Xu X. A Density-based Algorithm for Discovering Clusters a Density-based Algorithm for Discovering Clusters in Large Spatial Databases with Noise. Proceedings of the Second International Conference on Knowledge Discovery and Data Mining [Internet]. Portland, Oregon: AAAI Press; 1996. pp. 226–31. Available from: http://dl.acm.org/citation.cfm?id=3001460.3001507

6. Fraley C, Raftery AE. Model-Based Clustering, Discriminant Analysis, and Density Estimation. J Am Stat Assoc [Internet]. 2002;97:611–31. Available from: http://www.tandfonline.com/doi/abs/10.1198/016214502760047131

7. Thorndike RL. Who belongs in the family? Psychometrika [Internet]. Springer-Verlag; 1953;18:267–76. Available from: https://link.springer.com/article/10.1007/BF02289263

8. Rousseeuw PJ. Silhouettes: A graphical aid to the interpretation and validation of cluster analysis. J Comput Appl Math [Internet]. 1987;20:53–65. Available from: http://www.sciencedirect.com/science/article/pii/0377042787901257

9. Luxburg U von. Clustering Stability: An Overview. Foundations and Trends in Machine Learning [Internet]. Now Publishers; 2010;2:235–74. Available from: http://dx.doi.org/10.1561/2200000008

10. Ben-Hur A, Elisseeff A, Guyon I. A stability based method for discovering structure in clustered data. Pac Symp Biocomput [Internet]. 2002;6–17. Available from: https://www.ncbi.nlm.nih.gov/pubmed/11928511

11. Kiselev VY, Kirschner K, Schaub MT, Andrews T, Yiu A, Chandra T, et al. SC3: consensus clustering of single-cell RNA-seq data. Nat Methods [Internet]. 2017;14:483–6. Available from: http://dx.doi.org/10.1038/nmeth.4236

12. Rebane G, Pearl J. The Recovery of Causal Poly-Trees from Statistical Data. 2013; Available from: http://arxiv.org/abs/1304.2736

13. Csardi G, Nepusz T. The igraph software package for complex network research. InterJournal, Complex Systems. 2006;1695:1–9.

14. Reingold EM, Tilford JS. Tidier Drawings of Trees. IEEE Trans Software Eng [Internet]. 1981;SE-7:223–8. Available from: http://dx.doi.org/10.1109/TSE.1981.234519

15. Sugiyama K, Tagawa S, Toda M. Methods for Visual Understanding of Hierarchical System Structures. IEEE Trans Syst Man Cybern [Internet]. 1981;11:109–25. Available from: http://dx.doi.org/10.1109/TSMC.1981.4308636

16. Anderson E. The Irises of the Gaspe Peninsula. Bulletin of the American Iris Society. 1935;59:2–5.

17. Fisher RA. The use of multiple measurements in taxonomic problems. Ann Eugen [Internet]. Blackwell Publishing Ltd; 1936;7:179–88. Available from: http://dx.doi.org/10.1111/j.1469-1809.1936.tb02137.x

18. Tang F, Barbacioru C, Wang Y, Nordman E, Lee C, Xu N, et al. mRNA-Seq whole-transcriptome analysis of a single cell. Nat Methods [Internet]. 2009;6:377–82. Available from: http://dx.doi.org/10.1038/nmeth.1315

19. Stegle O, Teichmann SA, Marioni JC. Computational and analytical challenges in single-cell transcriptomics. Nat Rev Genet [Internet]. Nature Publishing Group; 2015;16:133–45. Available from: http://dx.doi.org/10.1038/nrg3833

20. Zheng GXY, Terry JM, Belgrader P, Ryvkin P, Bent ZW, Wilson R, et al. Massively parallel digital transcriptional profiling of single cells. Nat Commun [Internet]. 2017;8:14049. Available from: http://dx.doi.org/10.1038/ncomms14049

21. Satija R, Farrell JA, Gennert D, Schier AF, Regev A. Spatial reconstruction of single-cell gene expression data. Nat Biotechnol [Internet]. Nature Publishing Group; 2015;33:495–502. Available from: http://dx.doi.org/10.1038/nbt.3192

22. Wickham H. ggplot2: Elegant Graphics for Data Analysis [Internet]. Springer New York; 2010. Available from: https://market.android.com/details?id=book-rhRqtQAACAAJ

23. Blondel VD, Guillaume J-L, Lambiotte R, Lefebvre E. Fast unfolding of communities in large networks. J Stat Mech [Internet]. IOP Publishing; 2008;2008:P10008. Available from: http://iopscience.iop.org/article/10.1088/1742-5468/2008/10/P10008/meta
