# Supplementary material for: Clustering trees: a visualization for evaluating clusterings at multiple resolutions
Source: Gigascience. 2018 Jul 11;7(7):giy083. doi: 10.1093/gigascience/giy083 (PMC6057528; doi:10.1093/gigascience/giy083)
Supplement: Response_to_Reviewer_Comments_Original_Submission.pdf [file giy083_response_to_reviewer_comments_original_submission.pdf]

Author's Response To Reviewer Comments

Close

Reviewer #1
The authors in the manuscript try to answer an important and biologically relevant question. The manuscript is written well and the message is clearly explained. However, we have some concerns
and comments on the manuscript.

1. The presented method is conceptually equivalent to visualisation of hierarchical clustering, only applicable to other clustering methods. This should be made more clear in the text.

We have mentioned the relationship to hierarchical clustering in the paper and discussed the differences between this and clustering trees. While we accept the similarities between them we believe that clustering trees are significantly different, both in how they are constructed and how they would be used.

2. We think more datasets should be considered in the study.

We have added an additional section that uses five simulated datasets to illustrate what clustering trees would look like in different scenarios based on a suggestion from reviewer 3. We believe that this is useful in helping to explain the concepts presented in the paper. Adding more real datasets would provide extra examples but in our opinion would not convey the messages of the manuscript with more clarity.

3. Clustertree considers cluster stability measured across ks. Cluster stability is not a novel concept and the authors should include an brief overview of the existing literature on cluster stability in the introduction (e.g. Ben-Hur et al. 2002, Luxburg 2010) and explain how their method is different from the existing approaches.

Thank you for the suggestion and the references. We had added a paragraph that mentions the concept of cluster stability more generally.

4. In application to scRNAseq the elements of the clustering tree are methodologically very similar to the cluster stability index introduced in the SC3 package (https://www.nature.com/articles/nmeth.4236). It would be good to have a comparison of the two methods.

We had not considered the SC3 stability index before and there are indeed similarities, particularly as both clustering trees and the SC3 measure can be produced from just a set of clustering labels. We believe this measure could be useful for users and have implemented this method in the clustree package. The SC3 stability is now automatically calculated for each cluster and can be used to colour the nodes of the tree. Examples of this are included in the simulation section and the differences discussed.

5. (major) It is not obvious (at least for us) to understand from the clustering tree which k is the best. Even for a simple iris dataset it was hard for me to guess that k=3 is the right k. Maybe there are too many colours in the tree picture. Could the authors provide an

algorithmic approach to suggest the appropriate k(s) based on the tree perhaps in conjunction with some kind of metadata laid over the tree?

We intend clustering trees to be a tool that can help make the decision of which resolution to use, but not one that can provide a concrete suggestion. This could have been made clearer in the previous version and we have tried to do so in our revised text. Adding the simulation examples gives the reader a much clearer demonstration of what can happen to a clustering tree as a dataset becomes over-clustered. We have also tried to emphasise that clustering trees become more useful when combined with other metrics or domain knowledge and that they provide a new way to visualise this information across resolutions.

Reviewed by Tallulah Andrews and Vladimir Kiselev
Reviewer #2
The paper presents a new method to construct clustering trees for single-cell RNA-seq. While I recognize the task is very important due to the emerging importance of single-cell technologies, the proposed method only contains incremental improvements. Before addressing the following concerns I have, I would not recommend acceptance.

We do not believe the reader has understood the point of this paper at all which is why they are recommending a rejection. We are not presenting a new clustering method. Our direct responses to the points in this review are below but we do not believe this a suitable review for this work.

Main concerns:

1. Clarity. This paper proposed a simple clustering method for ScRNA-seq. However, the difference to many other clustering method (e.g., hierarchical clustering) is not clearly stated. The novelty is not clear to me.

We do not propose a new clustering method but instead a new method for visualising the results of existing clustering methods across resolutions. This is discussed in the paper. We also mention that clustering trees could be used in any field that makes use of clustering, not just scRNA-seq analysis.

2. Validity. The paper constructs a hierarchical clustering tree without considering the specific characters of sparsity and high dropouts of single-cell RNA-seq. Due to the existence of drop-out, traditional Euclidean/correlation metrics are not reliable (See "Visualization and analysis of single-cell RNA-seq data by kernel-based similarity learning", Nature Methods, 2017). However, this paper did not provide any specific solution to this problem. I am wondering why this method is particularly suitable for single-cell RNA-seq.

Our method is not designed specifically for scRNA-seq data and is in fact independent of any type of dataset or clustering method. As explained in our response to the previous point we propose a method for visualising clustering results, not a new clustering method.

3. Experiments. This paper applies the proposed methods on one simulation and one real PBMC dataset. However, no comparisons with other methods is provided. It is very hard to judge how well the proposed method is really performing. Visualization is also hard to judge. The lack of detailed experiments and comparisons is the main concern before

acceptance.

The submitted version of the manuscript did not consider any simulated datasets but provided examples based on the real iris and PBMC datasets. Simulated datasets have been added in the revised manuscript. We do not believe there is an existing visualisation that is directly comparable but we have included the SC3 stability index as an example of an existing cluster stability measure.

4. References: This paper is missing a few important references about single-cell anlaysis: For instance: "Revealing the vectors of cellular identity with single-cell genomics", Nature Biotech., 2016

As our paper is not specifically about scRNA-seq data or analysis we do not feel the need to reference all important papers in that field. We have provided an introduction to scRNA-seq data that is designed to help a general reader understand the PBMC dataset and why clustering would be useful in that setting. We believe this is sufficient for a technique that could be applied to many fields.
Reviewer #3:
Identification of the suitable number of clusters is an age-old question in clustering analysis. Standard methods for identifying the number of clusters make use of information about the 'tightness' of the clusters and the stability of the clusters with respect to some parameters. In this manuscript, Zappia and Oshlack present a new visualisation approach to explore the stability of cluster at different resolutions using a polytree visual representation, which allows for overlap of information of individual features and other external knowledge. This is an intuitive and powerful visualisation approach which I believe will be of widespread applications. I think this is a clever application of the hierarchical graph drawing technique. The manuscript is well written. I believe this manuscript is of value to the community.

However, I want to make the following suggestions:
Major:
- In figure 3 and figure 4, there are number of cases where a node has two parents. In almost all cases, the child node is placed under the parent node with the smallest node numbering instead of the node with the highest 'in-proportion' edge. For example, in Figure 4, the polytree has two nodes with two parent nodes. In both cases, the child node is placed below the parent node with the smaller 'in-proportion'. I thought it would make more sense to place them with the parent node with the higher 'in-proportion'.

We agree that this is a problem and it is the result of using existing layout algorithms which do not consider weight of edges in any way, sometimes resulting in layouts which seem to favour less important edges. We have addressed this by using only a subset of important edges (those with the greatest in-proportion for each node) to construct the layout. This simple modification is now the default setting in the clustree packages and results in more attractive tree which address the concerns you raise.

- Two 'positive' examples are described in the manuscript. I think it would be instructive to showcase what the resulting visualisation may look like if the clustering was performed on data with no or little underlying clustering structure. Could your visualisation identify 'bad' clustering results? For example, would the clustering tree of an entirely randomly generated data set looks differently from a data set with a strong clustering structure? A simulation study could be instructive here.

Thank you for the suggestion of adding a simulation study. We have added a new section to the paper that show some simulated scenarios. As you have suggested two of these are “null” examples including randomly generated uniform noise or a single cluster. We believe that these are instructive for the reader in showing what trees look like in different situations and how nodes and edges change as datasets are over-clustered.

- There are a number of graph drawing techniques for polytree, can the authors briefly review these methods and explain why the Reingold-Tilford or the Sugiyama layout was used?

These layout algorithms were chosen as they are the two methods designed for tree-like graphs available in the igraph package. We have added a paragraph to the manuscript that briefly explains how these algorithms work and why they were chosen.
Minor:
- It is important to point out that technically your 'tree' is a polytree, which is also called a directed acyclic graph. I do not object to calling it a 'tree' for simplicity throughout the manuscript, but I think it should be clearly noted in the introduction.
Thank you for introducing us to the idea of a polytree, this is not a term we had heard of before. You are correct that this is the graph structure produced by our algorithm and we have mentioned that in the text.

Close
