# Supplementary material for: Clustering trees: a visualization for evaluating clusterings at multiple resolutions
Source: Gigascience. 2018 Jul 11;7(7):giy083. doi: 10.1093/gigascience/giy083 (PMC6057528; doi:10.1093/gigascience/giy083)
Supplement: Response_To_Reviewer_Comments_Revision_1.pdf [file giy083_response_to_reviewer_comments_revision_1.pdf]

Author's Response To Reviewer Comments

Close

We have now made the changes to the formatting requested by the editor.

Close
