# Supplementary material for: Clustering trees: a visualization for evaluating clusterings at multiple resolutions
Source: Gigascience. 2018 Jul 11;7(7):giy083. doi: 10.1093/gigascience/giy083 (PMC6057528; doi:10.1093/gigascience/giy083)
Supplement: Reviewer_1_Report_(Original_Submission) -- Vladimir Kiselev — 3/16/2018 Reviewed [file giy083_reviewer_1_report_(original_submission).pdf]

Reviewer Report

Title: **Clustering trees: a visualisation for evaluating clusterings at multiple resolutions**

Version: **Original Submission** **Date:** 3/16/2018

Reviewer name: **Vladimir Kiselev**

Reviewer Comments to Author:

The authors in the manuscript try to answer an important and biologically relevant question. The manuscript is written well and the message is clearly explained. However, we have some concerns and comments on the manuscript.

1. The presented method is conceptually equivalent to visualisation of hierarchical clustering, only applicable to other clustering methods. This should be made more clear in the text.

2. We think more datasets should be considered in the study.

3. Clustertree considers cluster stability measured across ks. Cluster stability is not a novel concept and the authors should include an brief overview of the existing literature on cluster stability in the introduction (e.g. Ben-Hur et al. 2002, Luxburg 2010) and explain how their method is different from the existing approaches.

4. In application to scRNAseq the elements of the clustering tree are methodologically very similar to the cluster stability index introduced in the SC3 package (https://www.nature.com/articles/nmeth.4236). It would be good to have a comparison of the two methods.

5. (major) It is not obvious (at least for us) to understand from the clustering tree which k is the best. Even for a simple iris dataset it was hard for me to guess that k=3 is the right k. Maybe there are too many colours in the tree picture. Could the authors provide an algorithmic approach to suggest the appropriate k(s) based on the tree perhaps in conjunction with some kind of metadata laid over the tree?

Reviewed by Tallulah Andrews and Vladimir Kiselev

Methods

Are the methods appropriate to the aims of the study, are they well described, and are necessary controls included? Yes

Conclusions

Are the conclusions adequately supported by the data shown? Yes

Reporting Standards

Does the manuscript adhere to the journal's guidelines on [minimum standards of reporting?](#) Yes

Choose an item.

Statistics

Are you able to assess all statistics in the manuscript, including the appropriateness of statistical tests used? Yes, and I have assessed the statistics in my report.

Quality of Written English

Please indicate the quality of language in the manuscript: Acceptable

Declaration of Competing Interests

Please complete a declaration of competing interests, considering the following questions:

- Have you in the past five years received reimbursements, fees, funding, or salary from an organisation that may in any way gain or lose financially from the publication of this manuscript, either now or in the future?
- Do you hold any stocks or shares in an organisation that may in any way gain or lose financially from the publication of this manuscript, either now or in the future?
- Do you hold or are you currently applying for any patents relating to the content of the manuscript?
- Have you received reimbursements, fees, funding, or salary from an organization that holds or has applied for patents relating to the content of the manuscript?
- Do you have any other financial competing interests?
- Do you have any non-financial competing interests in relation to this paper?

If you can answer no to all of the above, write 'I declare that I have no competing interests' below. If your reply is yes to any, please give details below.

I declare that I have no competing interests

I agree to the open peer review policy of the journal. I understand that my name will be included on my report to the authors and, if the manuscript is accepted for publication, my named report including any attachments I upload will be posted on the website along with the authors' responses. I agree for my report to be made available under an Open Access Creative Commons CC-BY license

(http://creativecommons.org/licenses/by/4.0/). I understand that any comments which I do not wish to be included in my named report can be included as confidential comments to the editors, which will not be published.

I agree to the open peer review policy of the journal

To further support our reviewers, we have joined with Publons, where you can gain additional credit to further highlight your hard work (see: https://publons.com/journal/530/gigascience). On publication of this paper, your review will be automatically added to Publons, you can then choose whether or not to claim your Publons credit. I understand this statement.

Yes Choose an item.
