# Supplementary material for: Clustering trees: a visualization for evaluating clusterings at multiple resolutions
Source: Gigascience. 2018 Jul 11;7(7):giy083. doi: 10.1093/gigascience/giy083 (PMC6057528; doi:10.1093/gigascience/giy083)
Supplement: Reviewer_2_Report_(Original_Submission) -- Bo Wang — 04/11/2018 Reviewed [file giy083_reviewer_2_report_(original_submission).pdf]

Reviewer Report

Title: Clustering trees: a visualisation for evaluating clusterings at multiple resolutions

Version: Original Submission **Date:** 11 Apr 2018

Reviewer name: Bo Wang

Reviewer Comments to Author:

The paper presents a new method to construct clustering trees for single-cell RNA-seq. While I recognize the task is very important due to the emerging importance of single-cell technologies, the proposed method only contains incremental improvements. Before addressing the following concerns I have, I would not recommend acceptance.

Main concerns:

1. Clarity. This paper proposed a simple clustering method for ScRNA-seq. However, the difference to many other clustering method (e.g., hierarchical clustering) is not clearly stated. The novelty is not clear to me.
2. Validity. The paper constructs a hierarchical clustering tree without considering the specific characters of sparsity and high dropouts of single-cell RNA-seq. Due to the existence of drop-out, traditional Euclidean/correlation metrics are not reliable (See "Visualization and analysis of single-cell RNA-seq data by kernel-based similarity learning", Nature Methods, 2017). However, this paper did not provide any specific solution to this problem. I am wondering why this method is particularly suitable for single-cell RNA-seq.
3. Experiments. This paper applies the proposed methods on one simulation and one real PBMC dataset. However, no comparisons with other methods is provided. It is very hard to judge how well the proposed method is really performing. Visualization is also hard to judge. The lack of detailed experiments and comparisons is the main concern before acceptance.
4. References: This paper is missing a few important references about single-cell anlaysis: For instance:
"Revealing the vectors of cellular identity with single-cell genomics", Nature Biotech., 2016

Methods

Are the methods appropriate to the aims of the study, are they well described, and are necessary controls included? Yes

Conclusions

Are the conclusions adequately supported by the data shown? No

Reporting Standards

Does the manuscript adhere to the journal's guidelines on minimum standards of reporting? Yes

Choose an item.

Statistics

Are you able to assess all statistics in the manuscript, including the appropriateness of statistical tests used? There are no statistics in the manuscript.

Quality of Written English

Please indicate the quality of language in the manuscript: Needs some language corrections before being published

Declaration of Competing Interests

Please complete a declaration of competing interests, considering the following questions:

- Have you in the past five years received reimbursements, fees, funding, or salary from an organisation that may in any way gain or lose financially from the publication of this manuscript, either now or in the future?
- Do you hold any stocks or shares in an organisation that may in any way gain or lose financially from the publication of this manuscript, either now or in the future?
- Do you hold or are you currently applying for any patents relating to the content of the manuscript?
- Have you received reimbursements, fees, funding, or salary from an organization that holds or has applied for patents relating to the content of the manuscript?
- Do you have any other financial competing interests?
- Do you have any non-financial competing interests in relation to this paper?

If you can answer no to all of the above, write 'I declare that I have no competing interests' below. If your reply is yes to any, please give details below.

I declare that I have no competing interests.

I agree to the open peer review policy of the journal. I understand that my name will be included on my report to the authors and, if the manuscript is accepted for publication, my named report including any attachments I upload will be posted on the website along with the authors' responses. I agree for my report to be made available under an Open Access Creative Commons CC-BY license (http://creativecommons.org/licenses/by/4.0/). I understand that any comments which I do not wish to be included in my named report can be included as confidential comments to the editors, which will not be published.

I agree to the open peer review policy of the journal

To further support our reviewers, we have joined with Publons, where you can gain additional credit to further highlight your hard work (see: https://publons.com/journal/530/gigascience). On publication of this paper, your review will be automatically added to Publons, you can then choose whether or not to claim your Publons credit. I understand this statement. Yes

Yes Choose an item.
