# Supplementary material for: Clustering trees: a visualization for evaluating clusterings at multiple resolutions
Source: Gigascience. 2018 Jul 11;7(7):giy083. doi: 10.1093/gigascience/giy083 (PMC6057528; doi:10.1093/gigascience/giy083)
Supplement: Reviewer_3_Report_(Original_Submission) -- Joshua W. K. Ho, PhD — 4/18/2018 Reviewed [file giy083_reviewer_3_report_(original_submission).pdf]

Reviewer Report

Title: **Clustering trees: a visualisation for evaluating clusterings at multiple resolutions**

Version: **Original Submission** **Date:** 4/18/2018

Reviewer name: **Joshua W. K. Ho**

Reviewer Comments to Author:

Identification of the suitable number of clusters is an age-old question in clustering analysis. Standard methods for identifying the number of clusters make use of information about the 'tightness' of the clusters and the stability of the clusters with respect to some parameters. In this manuscript, Zappia and Oshlack present a new visualisation approach to explore the stability of cluster at different resolutions using a polytree visual representation, which allows for overlap of information of individual features and other external knowledge. This is an intuitive and powerful visualisation approach which I believe will be of widespread applications. I think this is a clever application of the hierarchical graph drawing technique. The manuscript is well written. I believe this manuscript is of value to the community.

However, I want to make the following suggestions:

Major:
- In figure 3 and figure 4, there are number of cases where a node has two parents. In almost all cases, the child node is placed under the parent node with the smallest node numbering instead of the node with the highest 'in-proportion' edge. For example, in Figure 4, the polytree has two nodes with two parent nodes. In both cases, the child node is placed below the parent node with the smaller 'in-proportion'. I thought it would make more sense to place them with the parent node with the higher 'in-proportion'.
- Two 'positive' examples are described in the manuscript. I think it would be instructive to showcase what the resulting visualisation may look like if the clustering was performed on data with no or little underlying clustering structure. Could your visualisation identify 'bad' clustering results? For example, would the clustering tree of an entirely randomly generated data set looks differently from a data set with a strong clustering structure? A simulation study could be instructive here.
- There are a number of graph drawing techniques for polytree, can the authors briefly review these methods and explain why the Reingold-Tilford or the Sugiyama layout was used?

Minor:
- It is important to point out that technically your 'tree' is a polytree, which is also called a directed acyclic graph. I do not object to calling it a 'tree' for simplicity throughout the manuscript, but I think it should be clearly noted in the introduction.

Methods

Are the methods appropriate to the aims of the study, are they well described, and are necessary controls included? Choose an item.

Conclusions

Are the conclusions adequately supported by the data shown? Choose an item.

Reporting Standards

Does the manuscript adhere to the journal's guidelines on [minimum standards of reporting?]() Choose an item.

Choose an item.

Statistics

Are you able to assess all statistics in the manuscript, including the appropriateness of statistical tests used? Choose an item.

Quality of Written English

Please indicate the quality of language in the manuscript: Choose an item.

Declaration of Competing Interests

Please complete a declaration of competing interests, considering the following questions:

- Have you in the past five years received reimbursements, fees, funding, or salary from an organisation that may in any way gain or lose financially from the publication of this manuscript, either now or in the future?
- Do you hold any stocks or shares in an organisation that may in any way gain or lose financially from the publication of this manuscript, either now or in the future?
- Do you hold or are you currently applying for any patents relating to the content of the manuscript?
- Have you received reimbursements, fees, funding, or salary from an organization that holds or has applied for patents relating to the content of the manuscript?
- Do you have any other financial competing interests?
- Do you have any non-financial competing interests in relation to this paper?

If you can answer no to all of the above, write 'I declare that I have no competing interests' below. If your reply is yes to any, please give details below.

I declare that I have no competing interests.

I agree to the open peer review policy of the journal. I understand that my name will be included on my report to the authors and, if the manuscript is accepted for publication, my named report including any attachments I upload will be posted on the website along with the authors' responses. I agree for my report to be made available under an Open Access Creative Commons CC-BY license (http://creativecommons.org/licenses/by/4.0/). I understand that any comments which I do not wish to be included in my named report can be included as confidential comments to the editors, which will not be published.

I agree to the open peer review policy of the journal

To further support our reviewers, we have joined with Publons, where you can gain additional credit to further highlight your hard work (see: https://publons.com/journal/530/gigascience). On publication of this paper, your review will be automatically added to Publons, you can then choose whether or not to claim your Publons credit. I understand this statement.

Yes Choose an item.
